# Isolation and Optimization of Aflatoxin B_1_ Degradation by Uniform Design and Complete Genome Sequencing of Novel Deep-Sea *Kocuria rosea* Strain 13

**DOI:** 10.3390/toxins15090520

**Published:** 2023-08-24

**Authors:** Jingying Wang, Qiqi Chen, Peisheng Yan, Chunming Dong, Zongze Shao

**Affiliations:** 1 School of Environment, Harbin Institute of Technology, Harbin 150090, China; wangjingying0721@163.com (J.W.);; 2 School of Marine Science and Technology, Harbin Institute of Technology (Weihai), Weihai 264209, China; 3 Key Laboratory of Marine Genetic Resources, Third Institute of Oceanography, Ministry of Natural Resources of PR China, Xiamen 361005, China; 4 State Key Laboratory Breeding Base of Marine Genetic Resources, Xiamen 361005, China; 5 Key Laboratory of Marine Genetic Resources of Fujian Province, Xiamen 350002, China; 6 Southern Marine Science and Engineering Guangdong Laboratory (Zhuhai), Zhuhai 519080, China

**Keywords:** aflatoxin B_1_ degradation, *Kocuria rosea*, uniform design, optimization, complete genome sequencing

## Abstract

Aflatoxin B_1_ is a natural carcinogenic mycotoxin. The biological detoxification of aflatoxin could result in less environmental pollution, more moderate conditions, and less impact on food and feed, and be more convenient than physical and chemical methods. In this study, strain 13 with aflatoxin B_1_ degradation activity (67.47 ± 1.44%) was isolated and identified as *Kocuria rosea*. A uniform design was applied to optimize the degradation activity using a software Data Processing System, and a quadratic polynomial stepwise regression model was selected to investigate the relationships between the degradation rate and five independent variables. Furthermore, the optimal degradation conditions (culture temperature of 30 °C, culture time of 4.2 days, seawater ratio of 100%, pH of 7.11, and inoculation dosage of 0.09%) were verified with a degradation rate of 88 ± 0.03%, which was well matched with the predicted value (92.97%) of the model. Complete genome sequencing of *Kocuria rosea*, conducted with a combination of Illumina and single-molecule real-time sequencing, was used to analyze the genomic features and functions of the strain, which were predicted by the annotation based on seven databases, and may provide insights into the potential of *Kocuria rosea*, as well as providing a reference for degradation gene and protein mining. These results indicate that *Kocuria rosea* strain 13 has the ability to degrade aflatoxin B_1_ efficiently, and it also has the potential to provide aflatoxin-degrading enzymes.

## 1. Introduction

Aflatoxins (AFs) are derivatives of dihydrofuranoxanadione; they have one benzopyrone and one difuran ring and are mainly produced by the genera of *Aspergillus* [1]. Naturally occurring aflatoxins classified as Group 1 have been evaluated by the International Agency for Research on Cancer (IARC) of the World Health Organization and are regarded as carcinogenic to humans [2]. The most carcinogenic aflatoxin is aflatoxin B_1_ (AFB_1_), and it has been found that the consumption of food contaminated with AFB_1_ causes immune suppression, deformity, gene mutagenesis, and carcinogenesis [3,4]. McMillan et al. reported that, when humans are exposed to AFB_1_ at a dose of 20–120 µg/kg body weight per day for 1–3 weeks, acute aflatoxin poisoning occurs, which can cause abdominal pain, emesis, and even death [5,6]. 

McMillan et al. stated that even chronic aflatoxin intoxication could cause hepatic carcinoma [6]. Therefore, knowing how to prevent and degrade aflatoxins to avoid exposure to humans and animals has become an increasingly urgent need due to the seriousness of aflatoxin toxicity, the widespread contamination of agricultural products, and the strictness of international standards of AFB_1_ in food and feed. 

Physical methods of AFB_1_ detoxification require complex and strict operating conditions, such as heating at high temperatures [7], adsorbing with sodium bentonite [8] and smectite [9], optical radiation with ultraviolet light [10], gamma radiation [11], and light pulses [12]. Moreover, chemical methods, such as citric acid [13], ozone [14], ammonia gas, or alkali refining [15], might irreversibly change the composition and flavor of the food. Both physical and chemical methods might cause loss of sensory and nutritional value of food and feed, and be difficult to use on a large scale. 

However, biological methods could have less environmental pollution, more moderate conditions, less impact on food and feed, and more convenience than physical and chemical methods. The biological detoxification of aflatoxin mainly includes plant extract detoxification, biosorption, bacterial degradation, and fungal degradation. Regarding plant extract detoxification, horse radish peroxidase from groundnut [16], seed extracts from the medical plant *Trachyspermum ammi* (L.) Sprague ex turrill [17], and leaf extracts from *Adhatoda vasica* Nees [18] have been reported to have an excellent degradation capacity to aflatoxins. For biosorption, Lactobacillus strains can bind aflatoxins with peptidoglycans on cell walls [19], and glucans with the helical molecular structure on cell walls of *Saccharomyces cerevisiae* can form a specific complementary structure when binding with AFB_1_ [20]. However, biosorption might be reversible and cannot essentially destroy the chemical structure of toxins. Therefore, bacterial degradation and fungal degradation have been studied to reduce toxicity permanently. There has been an increasing number of bacteria capable of aflatoxin degradation; they mainly belong to the phyla of *Actinobacteria* [21], *Firmicute* [22,23], *Proteobacteria* [24], *Bacteroidetes* [25], and *Myxococcota* [26]. Apart from single-strain degradation, it has also been reported that a microbial consortium can also degrade AFB_1_ with dominant strains, including *Geobacillus* and *Tepidimicrobium* [27]. Additionally, F_420_H_2_ from *Mycobacterium smegmatis* [28], extracellular enzymes from *Myxococcus fulvus* [26] and *Bacillus subtilis* [29], and intracellular enzymes from *Rhodococcus rhodochrous* [30] have been shown to have the ability to degrade AFB_1_. Moreover, AFB_1_-degrading enzymes have also been isolated from fungi [31], such as laccase from *Trametes versicolor* [32], Mn peroxidase from *Pleurotus ostreatus* [33], *Phanerochaete sordida* [34], or *Cladosporium uredinicola* [35], and enzymes from *Aspergillus niger* [36]. 

Most AFB_1_-producing and AFB_1_-degrading microorganisms are isolated from soil, plants, food, feed, crops [37], or animal waste [38]. However, there might be unknown aflatoxin-control microorganisms in the marine environment, since it has been reported that *Emericella venezuelensis*, which can produce aflatoxin, originated from the sea [39]. There has only been one aflatoxin-inhibiting strain from the deep sea reported so far that could inhibit the growth of aflatoxigenic fungi hypha and the generation of AFB_1_ [40]; nevertheless, no strains with AFB_1_ degradation properties from the deep sea have been elucidated. In this study, AFB_1_-degrading strain 13 was isolated from the deep sea and identified as *Kocuria rosea*. Additionally, degradation conditions were optimized through uniform design (UD), and a quadratic polynomial stepwise regression model was selected. Moreover, the complete genome of strain 13 was sequenced, and genome annotations were analyzed to gain insights into the genome functions of the strain.

## 2. Results and Discussion

### 2.1. Screening and Identification of Degrading Strain 13

**Figure 1:**
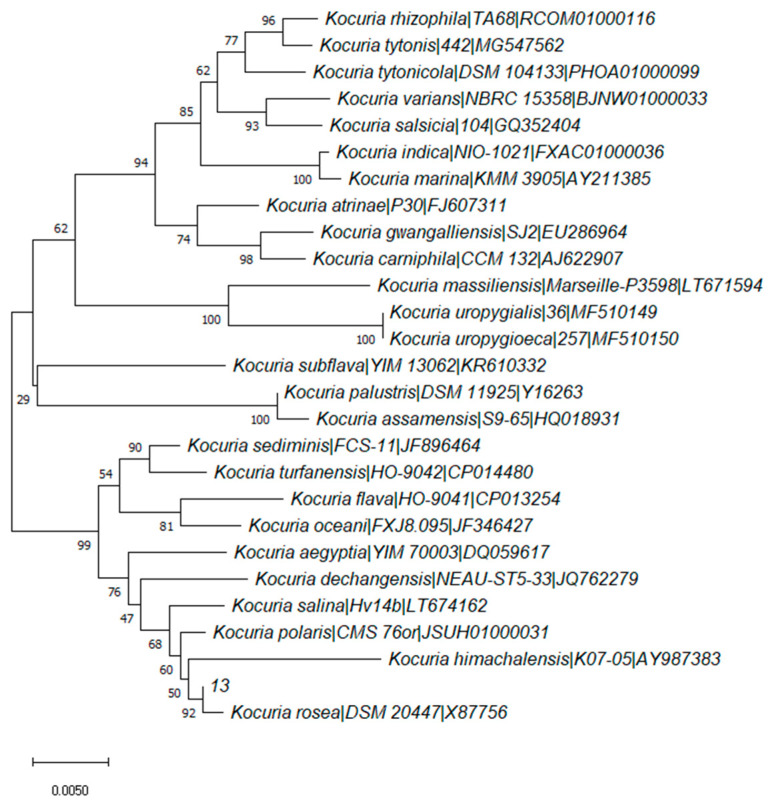
Phylogenetic tree for 27 Kocuria species based on 16S rRNA gene sequencing.

The strains were isolated from the colonies, and it was shown that strain 13 could degrade aflatoxin B_1_ with a rate of 67.47 ± 1.44% (with a culture temperature of 28 °C, culture time of 7 days, seawater ratio of 100%, pH of 7.52, and inoculation dosage of 1%) as optical density in 600nm (OD_600nm_) reached the value of 0.949 ± 0.016. The strain was identified according to a phylogenetic tree (Figure 1) by 16S rRNA gene sequencing, which shows the relationship of strain 13 between *Kocuria species* with other related strains. The similarity of strain 13 with type stain *Kocuria rosea* DSM 20447 was 99.65%, which indicates that stain 13 could be identified as *Kocuria rosea*. 

The biological functions of *Kocuria rosea* strains were discovered, including the biosorption and biomineralization of U (VI) [41], dyes degradation and decolorization (methyl orange, amido black, methyl violet, cotton blue, and malachite green) [42,43], phenol biodegradation [44], Keratin hydrolysis [45], trinitrotoluene detoxification [46], and polyaromatic hydrocarbons degradation [47,48,49]. Naphthalene, anthracene, phenanthrene, fluorene, and pyrene, degraded by *Kocuria rosea*, have at least two benzene rings and have a similar structure with aflatoxin B_1_, which indicates that Kocuria rosea strains have the potential to degrade substances containing benzene ring structures.

### 2.2. Optimization for AFB_1_ Degradation

**Figure 2:**
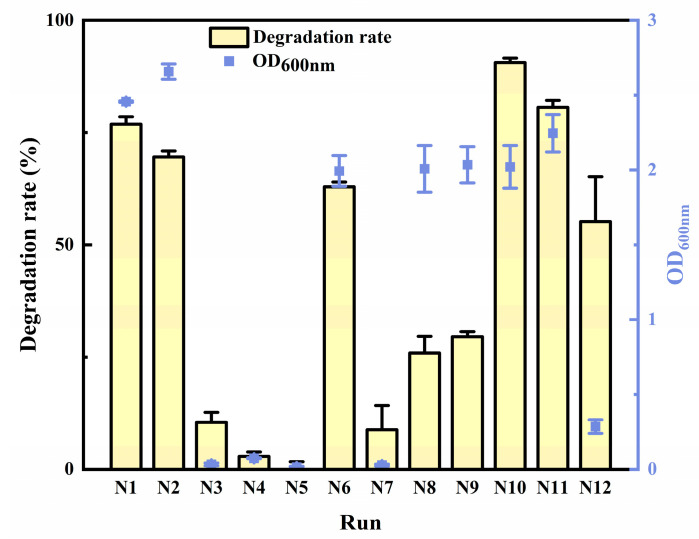
The degradation rate of uniform design experiments.

The results of the degradation rate from UD are shown in Figure 2. It was demonstrated that the degradation rate of N1, N2, N6, N10, and N11 with relatively high OD_600nm_ was over 60%. In order to select the model with a significant fitting effect, three types of quadratic polynomial mathematical models were evaluated and compared with the four parameters outlined in Table 1. The adjusted coefficient of determination ($R^{2}{}_{adj}$) represents the correlation between the observed values and the predicted values [50]. The closer $R^{2}{}_{adj}$ is to 1, the better the fitting effect achieved is. Root mean square error (RMSE) represents the differences between predicted and observed values and the precision of the predicted model [51,52]. Akaike’s information criterion (AIC) was derived from an asymptotic approximation to the Kullback−Leibler divergence between the true distribution and the model, and the Bayesian information criteria (BIC) derived from the dominant terms in the Laplace approximation to the logarithm of the Bayes factor with a vague prior [53]. Both AIC and BIC are two parameters commonly used for model selection, which were first introduced by Akaike [54] and Schwarz [55], respectively. However, the AIC assumes that the true model is not considered in all models, but the BIC assumes that the true model is one of the models [56]. The smaller the values of RMSE, AIC, and BIC of the models, the better the fitting effects which were demonstrated for the models were. The R^2^ of the quadratic polynomial stepwise regression model and stepwise regression model with multiple factors and interaction terms were much closer to one compared to the multivariate and squared stepwise regression model. Moreover, the RMSE, nAIC, and BIC of the quadratic polynomial stepwise regression model were the minimums in the three models. Therefore, it was indicated that the quadratic polynomial stepwise regression model had a better fitting effect than the other two models. 

The factors x2 × x3 and x2 × x2 in the quadratic polynomial stepwise regression model were removed for values of *p* greater than 0.05, which were 0.2404 and 0.2778, respectively. The formula of the model was generated as follows:

y = −1.349215599 + 0.13944838956 × x1 + 0.14664813874 × x2 − 0.003747153031 × x3 − 0.09323861795 × x5 − 0.0024620583619 × x1 × x1 + 0.00004951779435 × x3 × x3 + 0.009992701258 × x5 × x5 + 0.00023994501902 × x1 × x2 + 0.00005070750598 × x1 × x3 − 0.0014264859734 × x2 × x3

where y represents the degradation rate (%); x1 represents the culture temperature (°C); x2 represents the culture time (days); x3 represents the seawater ratio (%); x4 represents pH; and x5 represents the inoculation dosage (%).

**Figure 3:**
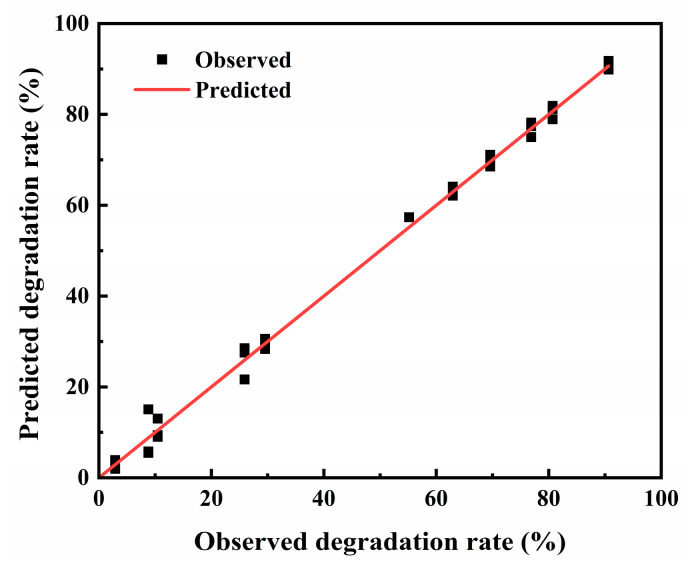
The degradation rate of predicted and observed values in uniform experimental design.

Multi-way analysis of variance (ANOVA) (Table 2) was applied in model evaluation, showing that the *p*-value was below 0.05, which indicated the great predictive ability of the model. The correlation coefficient (R), determinate coefficient (R^2^), and adjusted determinate coefficient (adj. R^2^) of the equation were 0.999, 0.999998, and 0.99978, respectively, which also indicated that the model could well reflect the relationship among culture temperature, culture time, seawater ratio, pH, and inoculation dosage. The relationship between the predicted values and observed values of the degradation rate are also confirmed in Figure 3, which shows that most points were distributed along a straight line, indicating that the predicted values and observed values were very close. Furthermore, a two-way ANOVA and multiple comparisons of least significant difference (LSD) for the predicted value and observed value were performed, demonstrating that the observed value of three replications (*p* = 0.988, 0.851, and 0.865 > 0.5) had no significant differences from the predicted value. Therefore, the quadratic polynomial stepwise regression model effectively estimated the degradation rate of strain 13 cultured under different conditions. 

**Figure 4:**
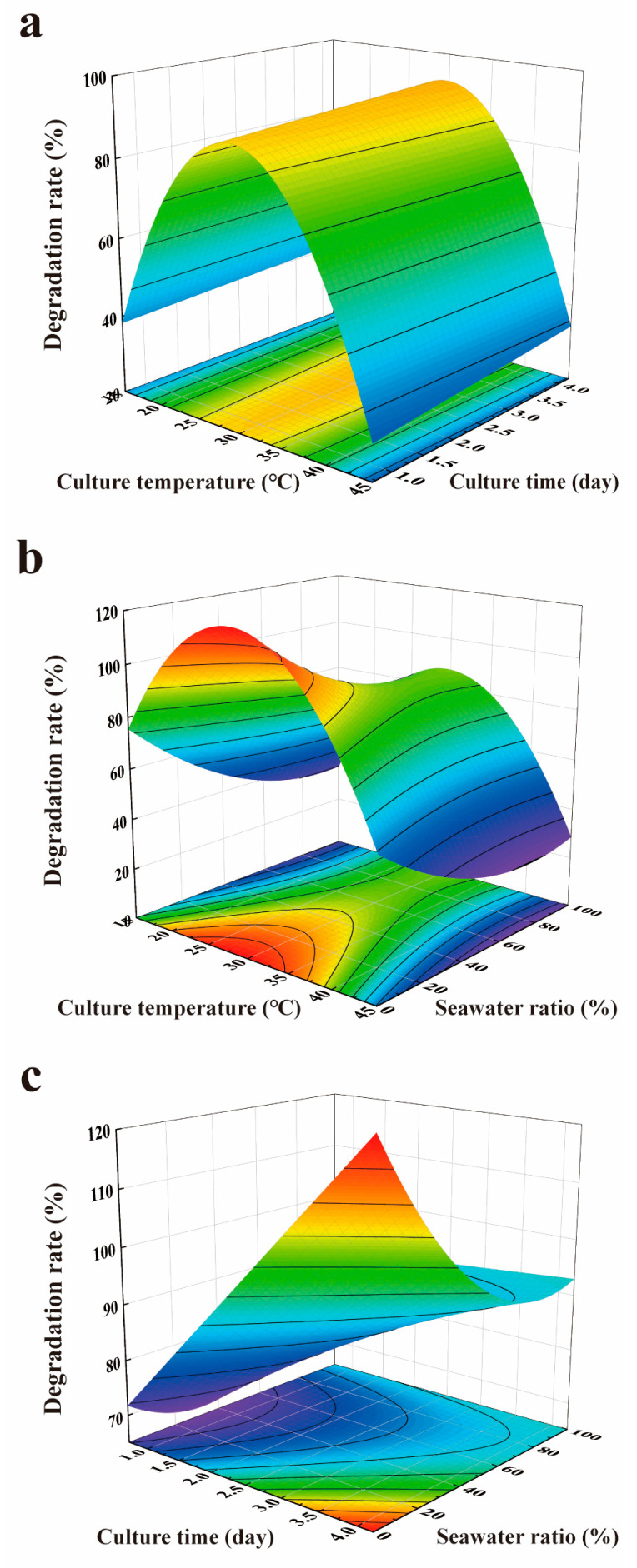
Response surface plots and contour plots of AFB_1_ degradation rate affected by interaction terms of two factors: (**a**) x1 − x2; (**b**) x1 − x3; (**c**) x2 − x3.

Parameter estimation and significance test of the model were performed. It was shown that the *p*-values of the ten factors were all less than 0.05, indicating that these ten factors significantly affected the degradation rate (Table 3). According to standard regression coefficients, factors x1, x3 × x3, x2, x1 × x3, x5 × x5, and x1 × x2 had, in descending order, significant positive effects on the degradation rate. Additionally, factors x1 × x1, x2 × x3, x3, and x5 had, in descending order, significant negative effects on the degradation rate. The predicted model for interaction terms varying within the experimental range was visualized through response surface plots and contour plots, and other variables remained at the optimal level (Figure 4). The optimal temperature was 30 °C no matter what the culture time and seawater ratio was, as shown in Figure 4a,b. The contribution of x1 (standard regression coefficient) to the equation was 4.86; however, the contribution of x1 × x2 and x1 × x3 was only 0.03 and 0.20, respectively (Table 3), which explained that x1 had a more significant influence in x1 × x2 and x1 × x3 than x2 and x3 (Figure 4a,b). It was also indicated that culture temperature had the most significant impact on the degradation rate compared to other factors (Table 3). Moreover, although x2 × x3 had an extremely negative effect (*p* < 0.01) on the degradation rate with a standard regression coefficient of −0.50, x2 had a significantly positive effect (*p* < 0.01) with a standard regression coefficient of 0.55. Consistently, it was observed that higher culture time was beneficial to the degradation rate (Figure 4c).

The optimal degradation conditions were predicted as a culture temperature of 30 °C, culture time of 4.2 days, seawater ratio of 100%, pH of 7.1094, and inoculation dosage of 0.0899%, with a degradation rate of 92.97%. Additionally, the confirmation experiments showed that the degradation rate was 88 ± 0.03%. Furthermore, the results of the single-sample *t*-test (t = −2.845; *p* = 0.105 > 0.05) showed that the original hypothesis (H0: µ = 92.97%) could not be rejected, which demonstrated that there were no significant differences between the predicted value and observed value.

### 2.3. General Genomic Features of Strain 13

**Figure 5:**
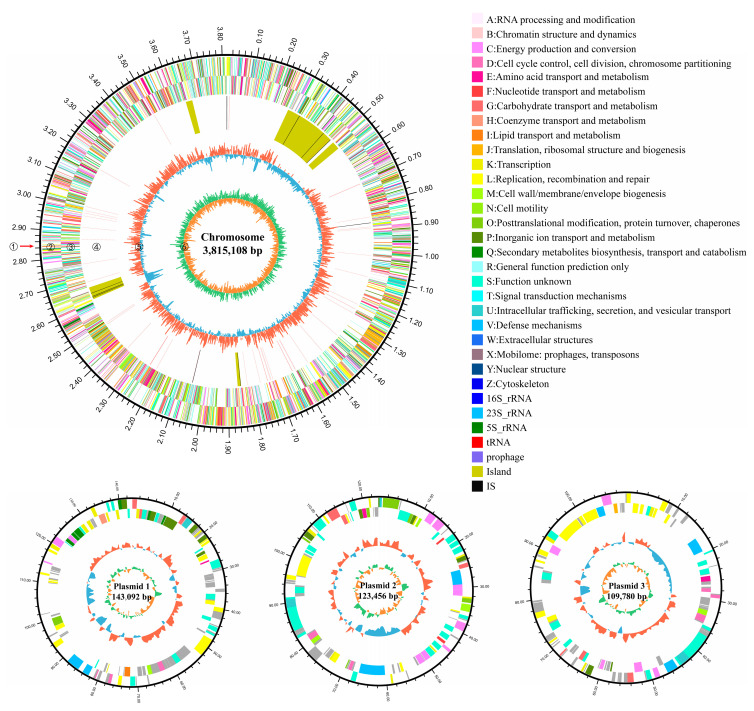
The circular genome map of strain 13 for one chromosome and three plasmids. Six circles in each map from the outermost circle to the innermost circle represent the following genome features: (1) gene size scale (each major scale mark representing 0.1 Mb), (2) CDS on forward chains with clusters of orthologous groups of proteins (COGs) categories in different colors, (3) CDS on reverse chains with COGs categories in different colors, (4) rRNA and tRNA, (5) guanine–cytosine (GC) content, (6) GC skew.

To better understand the AFB_1_ degradation mechanisms of strain 13, the complete genome of strain 13 was sequenced and mined. A graphical circular genome map of strain 13 is shown in Figure 5. The complete genome sequences of *Kocuria rosea* 13 were assembled into four scaffolds, including a chromosome and three plasmids. The chromosome had a size of 3,815,108 bp and a GC content of 72.86%. The predicted coding sequence has 3797 genes with a total length of 3,692,208 bp accounting for 91% of the complete genome, which also has 72.6% GC in the gene region. Additionally, there were 136 tandem repeats predicted with a ratio of 48% in the genome. Moreover, 91 RNA genes were predicted: 50 tRNA genes, 32 sRNA, and 9 rRNA genes, including 3 16S rRNA genes, 3 23S rRNA, and 3 5S rRNA genes. Regarding mobile genetic elements, six gene islands, eight clustered regularly interspaced short palindromic repeat (CRISPR)-Cas systems, and one prophage were predicted.

### 2.4. Gene Function Analysis

The CDS genome was annotated according to the following seven databases: Non-Redundant Protein Database (NR), Swiss-Prot, evolutionary genealogy of genes: Non-supervised Orthologous Groups (EggNOG), Pfam, Gene Ontology (GO), Kyoto Encyclopedia of Genes and Genomes (KEGG), and Carbohydrate-active enzymes (CAZy). The results of annotations for NR (Table S1), Swiss-Prot (Table S2), and Pfam (Table S3) were related to 3768, 2690, and 3120 genes, respectively.

**Figure 6:**
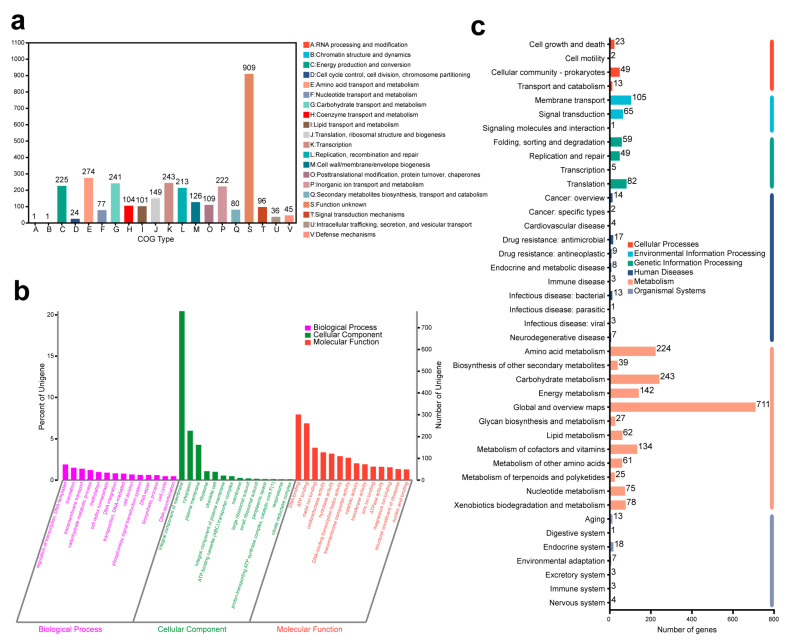
Histograms of function gene number: (**a**) EggNOG annotations; (**b**) GO annotations; (**c**) KEGG annotations.

EggNOG annotations divided 3231 genes, accounting for 85.09% of all the genes of strain 13 into 20 categories (Figure 6a). Type G Carbohydrate transport and metabolism had 241 genes which might be related to AFB_1_ degradation. Additionally, 80 genes annotated for secondary metabolites biosynthesis, transport, and catabolism could also be related to toxin degradation. Different gene numbers were counted from 1 to 274 for various function types; however, there were still unknown functions of 909 genes.

GO analysis classified 2698 genes (71.06% of all genes) from strain 13 into three major categories, including biological process (1149 genes), cellular component (1175 genes), and molecular function (2195 genes) (Figure 6b and Table S4). In the biological process, the GO annotations of top five genes were regulation of transcription and DNA-templated (GO ID: 0006355), translation (GO ID: 0006412), transmembrane transport (GO ID: 0055085), carbohydrate metabolic process (GO ID: 0005975), and methylation (GO ID: 0032259), which were related to 72 genes, 57 genes, 52 genes, 46 genes, and 37 genes, respectively. The biological process annotations of strain 13 contained 428 sub-functions, and molecular function annotations had 831 sub-functions. However, cellular component annotations had only 47 sub-functions. In the cellular component, the integral component of membrane (GO ID: 0016021) had the most genes (776 genes) in all GO annotations, and cytoplasm (GO ID: 0005737) and plasma membrane (GO ID: 0005886) had 227 genes and 162 genes, respectively. Furthermore, the carbohydrate metabolic process (GO ID: 0005975), oxidation-reduction process (GO ID: 0055114), aromatic amino acid family biosynthetic process (GO ID: 0009073), tetrahydrofolate metabolic process (GO ID: 0046653), aromatic compound catabolic process (GO ID: 0019439), mycothiol-dependent detoxification (GO ID: 0010127), and xenobiotic detoxification by transmembrane (GO ID: 1990961) might be related to AFB_1_ degradation.

In KEGG annotations, there were six primary categories (organismal systems, environmental information processing, human diseases, cellular processes, genetic information processing, and metabolism) of KEGG pathways corresponding to 1748 genes of strain 13, and each category contained different numbers of pathways (Figure 6c). Most numbers of genes (711 genes) were in connection with the global and overview maps in the largest category metabolism. Moreover, carbohydrate metabolism (243 genes), biosynthesis of other secondary metabolites (39 genes), and xenobiotics biodegradation and metabolism (78 genes) might be related to AFB_1_ degradation. 

Carbohydrate-active enzymes (CAZyme) contain auxiliary activities (AAs), carbohydrate-binding modules (CBMs), polysaccharide lyases (PLs), carbohydrate esterases (CEs), glycoside hydrolases (GHs), and glycosyl transferases (GTs) which could degrade, modify, and generate glycosidic bonds. To reveal the mechanism of the microbial carbohydrate metabolism, CAZy was used for the prediction and classification of CAZyme in stain 13. Only four types of CAZymes were identified from the complete genes of the strain, which were AAs (17 genes), CEs (22 genes), GHs (43 genes), and GTs (43 genes) (Table S5). There were the highest gene counts for enzymes in the families of GHs and GTs, which played a pivotal part in the degradation of polymers.

## 3. Conclusions

In summary, *Kocuria rosea* strain 13 was found to degrade AFB_1_ (88 ± 0.03%) in optimized conditions (culture temperature of 30 °C, culture time of 4.2 days, seawater ratio of 100%, pH of 7.1094, and inoculation dosage of 0.0899%). Therefore, this study suggests that *Kocuria rosea* could be used for aflatoxin degradation. Moreover, the annotations of the genome predicted the potential of the strain, and some genes might be related to degradation mechanisms, which could be further screened and verified by transcriptomics techniques in subsequent research. Future work could also focus on the identification and toxicity assessment of the degradation products metabolized by the strain.

## 4. Materials and Methods

### 4.1. Chemicals and Culture Media

AFB_1_ (purity > 99%) was purchased from J&K Scientific Technology (Beijing, China). The composition of the M2 medium was (1 L seawater) 0.5 g peptone, 0.5 g yeast extract, 0.5 g starch, 0.5 g sucrose, 0.5 g glucose, 5 g sodium acetate, 0.05 g potassium sodium tartrate, 0.05 g malic acid, 0.05 g trisodium citrate, 1.0 g ammonium nitrate, and 0.2 g ammonium chloride adjusted to pH 7.5~7.6. The MilliPore Synergy UV water purification system (Merck, Germany) was used to produce ultrapure water with resistivity in 18.2 MΩ-cm.

### 4.2. Isolation of the Strain 13

Coumarin was added into a tube as the solid substrate, and the tube was wrapped up with nylon mesh in a case consumed by deep-sea organisms. Additionally, sterilization was performed at 115 °C for 30 min. The wrappage was placed in sterilized incubation chambers (ICs) of the deep-sea in situ microbial incubator (DIMI), which was placed at a flat-topped seamount in the West Pacific Ocean (N 20.4059567°, E 160.7700883°) at 1617 m depth for 348 days of cultivation. The samples were collected and diluted tenfold with sterile seawater. The suspension was enriched for 20 days at 150 rpm with a shaker at 20 °C in an M2 liquid culture medium with 1 µg mL^−1^ AFB_1_. Additionally, 1 mL of the suspension was cultured with the same conditions for the second enrichment in an M2 seawater medium with 5 µg mL^−1^ AFB_1_. Then, the final enrichment cultures were diluted in a gradient, spread onto the M2 culture plate with AFB_1_ as the only carbon source, and cultured at 20 °C. To obtain pure stains, different colonies were picked, isolated, and incubated on M2 plates separately. The growth rate of the strains in the M2 liquid culture medium was monitored by a UV-2000 spectrophotometer (Unico, Shanghai, China) with OD_600nm_. As the strains grew and OD_600nm_ reached about 1.0, the suspensions were inoculated into an M2 liquid medium (AFB_1_ as the only carbon source) and cultured for seven days at 28 °C.

### 4.3. Determination of Aflatoxin Degradation Rate

AFB_1_ in the control groups and samples was extracted three times using chloroform with an equal volume before the solvent evaporated under N_2_ at room temperature [23,26,57,58]. Dimethyl sulfoxide (DMSO) (50 µL) was used to dissolve the dried extracts, and 20 µL of the mixture was injected in UltiMateTM3000 HPLC (Thermo Scientific, Bremen, Germany). HPLC analysis was conducted with a C18 Polaris column (250 mm × 4.6 mm i.d., 5 µm) in a mobile phase of water and methanol in a 1:1 ratio (*v*/*v*). The flow rate was set as 1 mL min^−1^, and a UV/VIS detector (Thermo Scientific, Germany) was used for absorbance measurements at a wavelength of 360 nm. The column temperature was set to 35 °C for detection. The software Chromeleon v6.8 was used for data analysis. The rate of AFB_1_ degradation was determined and calculated with (1 − AFB_1_ peak area in treatment/AFB_1_ peak area in control) × 100%.

### 4.4. UD for Aflatoxin Degradation Optimization

Aflatoxin degradation by the strain supernatant was optimized under UD according to [59]. Five independent variables were selected as follows: culture temperature (x1) (°C), culture time (x2) (days), seawater ratio (x3) (%), pH (x4), and inoculation dosage (x5) (%) (Table 4). Multiple mixed-level uniform designs U_12_ (4^1^ × 6^4^) were obtained with the software Data Processing System (DPS) 18.10 [60] by varying the parameters of random seed number, maximum iterations, and optimal search time. Seven UD matrix performance parameters [59] were compared among different UD tables, resulting in a UD (Table 5) with the smallest values of parameters being selected for the experimental scheme.

To assess degradation performance, the degradation rate was used as the dependent variable. The dependent variable could be related to the above five independent variables through three quadratic polynomial mathematical models: a quadratic polynomial stepwise regression model, a stepwise regression model with multiple factors and interaction terms, or a multivariate and squared stepwise regression model. The models were described using Equations (1)–(3).

Quadratic polynomial stepwise regression model:

(1) $$y=b_{0}+\sum_{i=1}^{m}b_{i}x_{i}+\sum_{i=1}^{m}b_{ii}x_{i}^{2}+\sum_{j=1}^{i<j}b_{ij}x_{i}x_{j}$$

Stepwise regression model with multiple factors and interaction terms:

(2) $$y=b_{0}+\sum_{i=1}^{m}b_{i}x_{i}+\sum_{i=1}^{i<j}b_{ij}x_{i}x_{j}$$

Multivariate and squared stepwise regression model:

(3) $$y=b_{0}+\sum_{i=1}^{m}b_{i}x_{i}+\sum_{i=1}^{m}b_{i}x_{i}^{2}$$

where y represents the dependent variable to be modeled; $x_{i}$ and $x_{j}$ represent the independent variables; $b_{ij}$, $b_{ii}$, $b_{i}$, and $b_{0}$ represent the interaction coefficients, quadratic coefficients, linear coefficient, and constant coefficient, respectively. 

The fit goodness of the three models was evaluated and compared using $R^{2}{}_{adj}$, RMSE, nAIC, and BIC. The equations of $R^{2}{}_{adj}$, RMSE, nAIC, and BIC can be described as follows:

(4) $$R^{2}{}_{adj}=1-\left(\frac{N-1}{N-n_{p}}\right)\frac{\sum_{i=1}^{n}{\left(O_{i}-P_{i}\right)}^{2}}{\sum_{i=1}^{n}{\left(O_{i}-m\right)}^{2}}$$

(5) $$RMSE=\sqrt{\frac{RSS}{N}}=\sqrt{\frac{\sum {\left(O_{i}-P_{i}\right)}^{2}}{N}}$$

(6) $$\mathrm{nAIC}=\ln\left(\frac{\sum {\left(O_{i}-P_{i}\right)}^{2}}{N}\right)+\frac{2*n_{p}}{N}$$

(7) $$\mathrm{BIC}=N*\ln\left(\frac{\sum^{\text{​}}{\left(O_{i}-P_{i}\right)}^{2}}{N}\right)+N*\left(n_{y}*\ln\left(2\pi \right)+1\right)+n_{p}*\ln N$$

where $O_{i}$ represents the *i*th measured observed value; $P_{i}$ represents the *i*th predicted value; *m* represents the average value; RSS represents the residual sum of squares; *N* represents the number of values in the estimation data set; *n_p_* represents the number of estimated parameters; and *n_y_* represents the number of model outputs.

### 4.5. DNA Extraction, Amplification, and 16S rRNA Gene Sequencing

The DNA of aflatoxin-degrading bacteria was extracted via a boiling lysis method: a single colony was selected and boiled in a 100 °C water bath for 15 min, then placed at 4 °C for 30 min, spun at 5000 rpm for one minute, and the extracted DNA in the supernatant was used for PCR amplification. An initial denaturation at 94 °C for 5 min, followed by 30 cycles of denaturation at 94 °C for 40 s, primer annealing at 55 °C for 40 s, extension for 1 min at 72 °C, and a final extension at 72 °C for 10 min, was used to amplify the DNA sample in a reaction mixture of 50 µL. The PCR product was purified and sent to Sangon Biotech Co., Ltd. (Shanghai, China) for sequencing. The sequences of the strain were assembled with the software DNAMAN 9.0.1 and aligned in EZTaxon (https://www.ezbiocloud.net/; accessed on 16 January 2023). The phylogenetic tree of the strain was constructed with the software MEGA 10.2.5 using the neighbor-joining (NJ) method. The bootstrap method replications were set as 1000. The 16S rDNA sequence of strain 13 (1521 bp) uploaded to GenBank was registered for the accession number CP127857. 

### 4.6. Complete Genome Sequencing of Kocuria rosea

#### 4.6.1. DNA Extraction

The bacterial cells were cultured to the logarithmic growth phase and collected via centrifuge CR21N (HITACHI, Tokyo, Japan) for 5 min at 14,000 rpm. The genomic DNA was extracted and purified with the Wizard^®^ genomic DNA purification kit (Promega Corp., Madison, WI, USA), and the purity and concentration were detected using agarose gel electrophoresis and Nanodrop 2000 (Thermo Scientific, Germany), respectively.

#### 4.6.2. Genomic Library Construction and Sequencing

The genome of the strain was sequenced with both Illumina sequencing and single-molecule real-time sequencing (SMRT). The Illumina data were used to assess genomic heterozygosity, genomic size, genomic duplication, presence of plasmids, and contamination, in addition to correcting long sequences from the third generation of sequencing to ensure the completeness and accuracy of the assembly.

For the Illumina platform, a focused acoustic shearer Covaris M220 (Covaris, Woburn, MA, USA) was used to shear DNA in a 1 µg genomic sample into 400–500 bp fragments. Additionally, the NEXTflex™ Rapid DNA-Seq kit (BIOO Scientific Co., Austin, TX, USA) and Illumina HiSeq X Ten (Illumina, San Diego, CA, USA) were used for library preparation and paired-end sequencing (2 × 150 bp), respectively.

For the SMRT platform, genomic DNA in a 15 µg sample was sheared into 8-10 kb fragments by a centrifuge 5424 (Eppendorf, Hamburg, Germany) at 6000 rpm for 1 min with a G-tube (Covaris, America). Both ends of the purified single-strand DNA fragments were connected with a sequencing adapter named SMRT bell for library construction. The Agencourt AMPure XP kit (Beckman Coulter Genomics, Woollahra, NSW, Australia) with 0.45× volumes was applied for library purification three times, and the library was sequenced by a PacBio RS II (Pacific Biosciences of California Inc., Menlo Park, CA, USA).

#### 4.6.3. Genome Assembly and Plasmid Identification

The complete genome sequences, including plasmids, were assembled with the reads of both Illumina and PacBio. The Illumina raw data saved as a FASTQ file were trimmed, and low-quality reads were removed for clean data. The software Unicycler v0.4.8 [61] was used for the assembly of the PacBio reads before the reads were corrected according to Illumina reads with Pilon v1.22 for the complete genome, including chromosome and plasmid sequence. In addition, PlasFlow (https://github.com/smaegol/PlasFlow; accessed on 16 January 2023) was used for plasmid identification. Furthermore, the plasmid sequences were annotated using the basic local alignment search tool (BLAST) and database PLSDB (https://ccb-microbe.cs.uni-saarland.de/plsdb/; accessed on 16 January 2023). The genome sequences were stored at GenBank with the accession numbers CP127857 (chromosome), CP127858 (plasmid A), CP127859 (plasmid B), and CP127860 (plasmid C).

#### 4.6.4. Structural Genomics Analysis

Glimmer v3.02, GeneMarkS v4.3, and Prodigal v2.6.3 were used for the prediction of the chromosome genome, plasmid genome, and codon sequence. Tandem repeats were identified with the Tandem Repeat Finder v4.04. Moreover, tRNA and rRNA were predicted with tRNAscan-SE v2.0 [62] and Barrnap, respectively. The sRNA was predicted using the software Infernal 1.1.3 (http://eddylab.org/infernal/, accessed on 16 January 2023) compared to the Rfam database (https://rfam.xfam.org/; accessed on 16 January 2023). The genomic island was predicted with Island Viewer [63], and CRISPRs were recognized with MinCED v3.0 (https://github.com/ctSkennerton/minced, accessed on 16 January 2023). Additionally, PHAST (http://phast.wishartlab.com/index.html; accessed on 16 January 2023) was used to search for possible prophage sequence. Genome visualization was displayed with Circos v0.69-6 (http://www.circos.ca, accessed on 16 January 2023) [64].

#### 4.6.5. Genome Function Annotation

The CDS genome was compared and annotated with different databases using the following software: Diamond v0.8.35 for NR of the National Center for Biotechnology Information (NCBI), Swiss-Prot [65], and EggNOG v4.5.1 [66]; HMMER v3.1b2 (http://www.hmmer.org/, accessed on 16 January 2023) for Pfam (http://pfam.xfam.org/, accessed on 16 January 2023) [67]; Blast2go v2.5 for GO; BLAST+ v2.3.0 for KEGG; Diamond v0.8.35 for CAZy (http://www.cazy.org/, accessed on 16 January 2023).

## Figures and Tables

**Table 1:** Parameters of model fitness.

Model	R^2^	RMSE	nAIC	BIC
The quadratic polynomial stepwise regression model	1.000	0.000	−19.728	25.918
The stepwise regression model with multiple factors and interaction terms	1.000	0.000	−18.198	27.448
The multivariate and squared stepwise regression model	0.954	0.055	−4.978	40.667

**Table 2:** Analysis of variance regression model parameters.

Source of Variation	Sum of Squares	Degree of Freedom	Mean Square	F-Value	*p*-Value
Regression	1.232811	10	0.123281	8,738,194	0.000263
Residual	1.41 × 10^−8^	1	1.41 × 10^−8^		
Total variation	1.232811	11			

**Table 3:** Parameter estimation and significance test of the model.

Factor	Regression Coefficient	Standard Regression Coefficient	Partial Correlation Coefficient	t-Value	*p*-Value
x1	0.139448	4.864194	1	1502.222	0.000424
x2	0.146648	0.546966	1	1178.142	0.00054
x3	−0.00375	−0.39932	−1	390.2854	0.001631
x5	−0.09324	−0.34776	−0.99999	264.1455	0.00241
x1 × x1	−0.00246	−5.20978	−1	2130.127	0.000299
x3 × x3	4.95 × 10^−5^	0.549742	1	1308.839	0.000486
x5 × x5	0.009993	0.186559	0.999963	116.7742	0.005452
x1 × x2	0.00024	0.031926	0.999878	63.88512	0.009964
x1 × x3	5.07 × 10^−5^	0.195935	0.999978	149.4907	0.004259
x2 × x3	−0.00143	−0.49983	−1	1134.843	0.000561

**Table 4:** The levels of five independent variables.

Independent Variables		1	2	3	4	5	6
Temperature (°C)	x1	15	25	35	45		
Time (Days)	x2	0.7	1.4	2.1	2.8	3.5	4.2
Seawater ratio (%)	x3	0	20	40	60	80	100
pH	x4	6	7	8	9	10	11
Inoculation dosage (%)	x5	0.7	1.4	2.1	2.8	3.5	4.2

**Table 5:** The scheme and the matrix performance parameters of the uniform design.

Run	x1	x2	x3	x4	x5	Uniform Design Matrix Performance Parameters
N1	2	3	2	3	1	Centered discrepancy = 0.18836
N2	2	6	5	1	4	L2-discrepancy = 0.03101
N3	4	3	6	3	6	Modified discrepancy = 0.26946
N4	1	1	4	2	5	Symmetric discrepancy = 1.06848
N5	4	2	3	1	2	Wrap-around discrepancy = 0.37733
N6	2	2	1	5	4	Design matrix condition number = 1.5899
N7	4	4	2	4	5	D-optimal = 0.0000
N8	1	5	3	6	6	
N9	1	4	6	4	2	
N10	3	5	1	2	3	
N11	3	6	4	5	1	
N12	3	1	5	6	3	

## Data Availability

The data presented in this study are available in this article.

## Supplementary Materials

The following supporting information can be downloaded at: https://www.mdpi.com/article/10.3390/toxins15090520/s1, Table S1: NR annotations; Table S2: SwissProt annotations; Table S3: Pfam annotations; Table S4: GO annotations; Table S5: CAZy annotations.
